# 2,3-Dihydro-1*H*-pyrrolo­[1,2-*a*]indole-9-carbonitrile

**DOI:** 10.1107/S1600536812045345

**Published:** 2012-11-10

**Authors:** Lee G. Madeley, Andreas Lemmerer, Joseph P. Michael

**Affiliations:** a Molecular Sciences Institute, School of Chemistry, University of the Witwatersrand, Private Bag 3, PO WITS, 2050, Johannesburg, South Africa

## Abstract

The asymmetric unit of the title compound, C_12_H_10_N_2_, which may serve as a model for mitosenes, contains two independent mol­ecules. The conformation of the five-membered rings in both molecules is envelope, with the central CH_2_—CH_2_—CH_2_ C atom at the flap in each case. In the crystal, they inter­act by a combination of weak C—H⋯N and π–π inter­actions [centroid–centroid distances = 3.616 (1) and 3.499 (1) Å] and C—H⋯π contacts.

## Related literature

For the synthesis of the title compound by intra­molecular Heck reaction of [1-(2-bromo­phen­yl)pyrrolidin-2-yl­idene]-acetonitrile, see: Michael *et al.* (1993 ▶). For an alternative synthesis by cyclization of [2-(2-oxopyrrolidin-1-yl)phen­yl]acetonitrile with sodium hydride, see: Verboom *et al.* (1986 ▶). For background to mitosenes, see: Franck (1978 ▶); Kasai & Kono (1992 ▶).
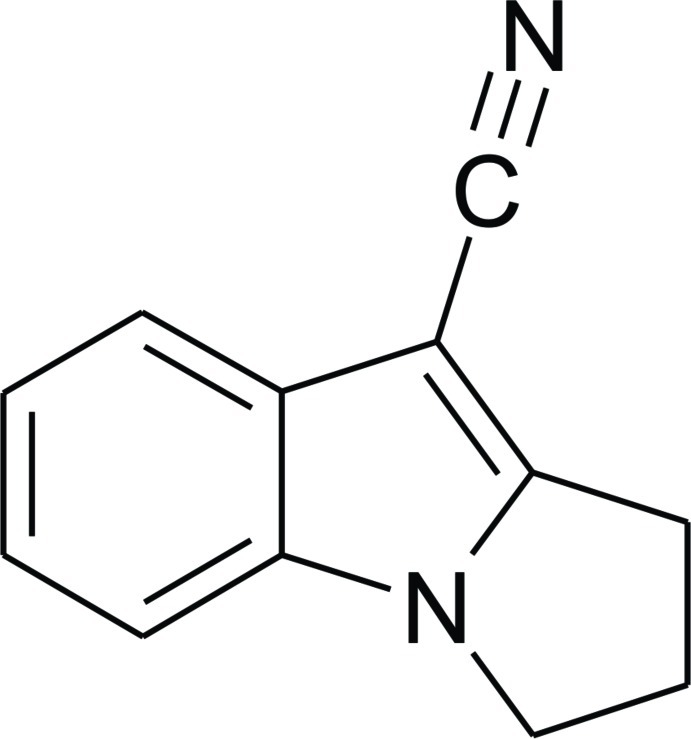


## Experimental

### 

#### Crystal data

C_12_H_10_N_2_
*M* *_r_* = 182.22
Triclinic, 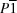

*a* = 9.1383 (3) Å
*b* = 9.5340 (3) Å
*c* = 12.3138 (4) Å
α = 90.794 (2)°
β = 90.528 (2)°
γ = 116.272 (2)°
*V* = 961.78 (5) Å^3^
*Z* = 4
Mo *K*α radiation
μ = 0.08 mm^−1^
*T* = 173 K
0.50 × 0.45 × 0.30 mm

#### Data collection

Bruker APEXII CCD area-detector diffractometer
Absorption correction: multi-scan (*SADABS*; Sheldrick, 1996 ▶) *T*_min_ = 0.963, *T*_max_ = 0.978
7592 measured reflections
3498 independent reflections
2809 reflections with *I* > 2σ(*I*)
*R*_int_ = 0.023

#### Refinement

*R*[*F* ^2^ > 2σ(*F* ^2^)] = 0.038
*wR*(*F* ^2^) = 0.097
*S* = 1.04
3498 reflections
254 parameters
H-atom parameters constrained
Δρ_max_ = 0.19 e Å^−3^
Δρ_min_ = −0.15 e Å^−3^

### 

Data collection: *APEX2* (Bruker, 2005 ▶); cell refinement: *SAINT-Plus* (Bruker, 2004 ▶); data reduction: *SAINT-Plus* and *XPREP* (Bruker 2004 ▶); program(s) used to solve structure: *SHELXS97* (Sheldrick, 2008 ▶); program(s) used to refine structure: *SHELXL97* (Sheldrick, 2008 ▶); molecular graphics: *ORTEP-3 for Windows* (Farrugia, 1997 ▶) and *DIAMOND* (Brandenburg, 1999 ▶); software used to prepare material for publication: *WinGX* (Farrugia, 1999 ▶) and *PLATON* (Spek, 2009 ▶).

## Supplementary Material

Crystal structure: contains datablock(s) global, I. DOI: 10.1107/S1600536812045345/bh2462sup1.cif

Supplementary material file. DOI: 10.1107/S1600536812045345/bh2462Isup2.mol

Structure factors: contains datablock(s) I. DOI: 10.1107/S1600536812045345/bh2462Isup3.hkl

Supplementary material file. DOI: 10.1107/S1600536812045345/bh2462Isup4.cml

Additional supplementary materials:  crystallographic information; 3D view; checkCIF report

## Figures and Tables

**Table 1:** Hydrogen-bond geometry (Å, °) *Cg*1 and *Cg*2 are the centroids of the C6*A*–C11*A* and C6*B*–C11*B* rings, respectively.

*D*—H⋯*A*	*D*—H	H⋯*A*	*D*⋯*A*	*D*—H⋯*A*
C1*A*—H1*A*1⋯N2*B*	0.99	2.68	3.338 (2)	124
C2*A*—H2*A*1⋯N2*B*	0.99	2.66	3.373 (2)	129
C3*A*—H3*A*2⋯N2*A* ^i^	0.99	2.66	3.634 (2)	168
C3*B*—H3*B*1⋯N2*B* ^ii^	0.99	2.57	3.495 (2)	156
C3*A*—H3*A*1⋯*Cg*1^iii^	0.99	2.79	3.545 (2)	135
C3*B*—H3*B*2⋯*Cg*2^iv^	0.99	2.67	3.523 (2)	146

Symmetry codes: (i) 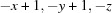; (ii) 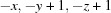; (iii) 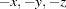; (iv) 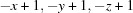.

**Fig. 1.**
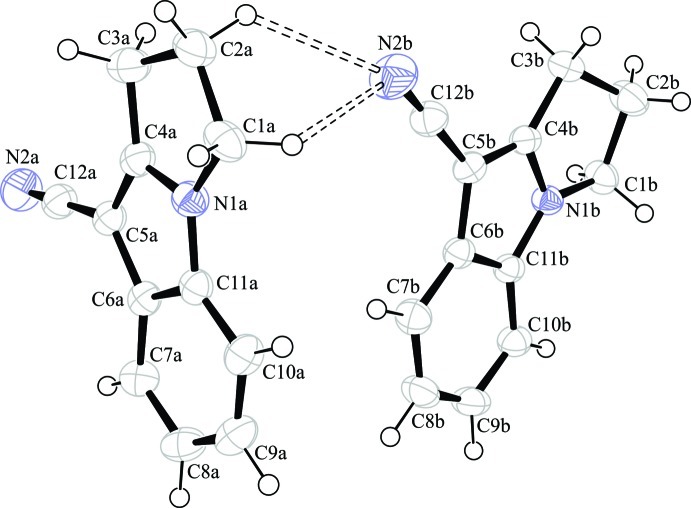
The asymmetric unit of (I) showing the atomic numbering scheme. Displacement ellipsoids are shown at the 50% probability level.

**Fig. 2.**
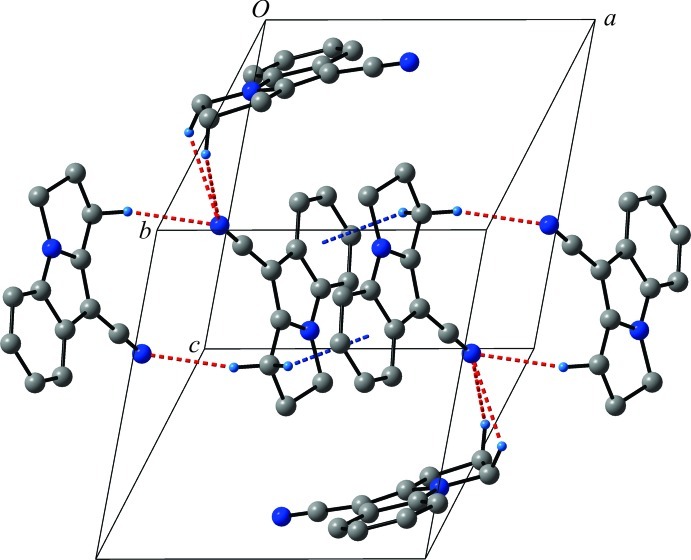
View of the hydrogen bonds of (I). C—H···N are shown as dashed red lines and C—H···π as dashed blue lines. H atoms not involved in hydrogen bonding are omitted for clarity.

## supplementary crystallographic information

### Comment

The mitosenes, naturally occurring biologically active degradation products of the important mitomycin antibiotics (Franck, 1978; Kasai & Kono, 1992) are characterized by the presence of a pyrrolo[1,2-*a*]indole core. The title compound, 2,3-dihydro-1*H*-pyrrolo[1,2-*a*]indole-9-carbonitrile, was prepared as part of a model study on the use of intramolecular Heck reactions for creating this core from various [1-(2-bromoaryl)pyrrolidin-2-ylidene]acetates and analogues (Michael *et al.*, 1993).

The asymmetric unit of (I) consists of two molecules, labelled *A* and *B*, on general positions. Fig. 1 shows the atomic numbering scheme. The hydrogen bonding of (I) consists of weak C—H···N hydrogen bonds and various π–π interactions. Each molecule in the asymmetric unit makes centrosymmetric dimers using the C3*A*—H3*A*2···N2*A* and C3*B*—H3*B*1···N2*B* hydrogen bonds, shown explicitly for the *B* molecule in Fig. 2. Between the *A* and *B* molecules, two ethylene groups from the *A* molecule hydrogen bond to the cyanide N atom of the *B* molecule, through C1*A*—H1*A*1···N2*B* and C2*A*—H2*A*1···N2*B* hydrogen bonds. In addition, both *A*/*A* and *B*/*B* molecules sit parallel to each other and undergo π–π interactions, with distances of 3.616 (1) Å for *A*···*A* and 3.499 (1) Å for *B*···*B* (Fig. 2). Also C—H···π contacts are formed between those same *A*/*A* and *B*/*B* molecules, C3*A*—H3*A*1···*Cg*1*^iii^* [*Cg*1: C6*A* to C11*A*; symmetry operator: (*iii*) -*x*, -*y*, -*z*] and C3*B*—H3*B*2···*Cg*2*^iv^* [*Cg*2: C6*B to* C11*B*; symmetry operator: (*iv*) 1-*x*, 1-*y*, 1-*z*].

### Experimental

The title compound was prepared by reaction of [1-(2-bromophenyl)pyrrolidin-2-ylidene]acetonitrile (350 mg, 1.33 mmol) with palladium(II) acetate (299 mg, 1.33 mmol, 1 eq.), tri-*o*-tolylphosphine (407 mg, 1.33 mmol) and triethylamine (0.19 ml, 1.33 mmol), heated under reflux in acetonitrile (7 ml) for 96 h. The crude oil obtained after evaporation of the solvent (1.20 g) was purified by column chromatography on silica gel with hexane/ethyl acetate (5:1 *v*/*v*) as eluent to yield a colourless solid (133 mg, 55%). Recrystallization from ethyl acetate produced colourless blocks, m.p. 400–401 K. An alternative synthesis is available from the literature, based on cyclization of [2-(2-oxopyrrolidin-1-yl)phenyl]acetonitrile with sodium hydride (Verboom *et al.*, 1986).

### Refinement

The C-bound H atoms were geometrically placed (C—H bond lengths of 0.95 for aromatic CH and 0.99 for methylene CH_2_) and refined as riding with *U*_iso_(H) = 1.2*U*_eq_(C).

### Crystal data

**Table:**

C_12_H_10_N_2_	*Z* = 4
*M**_r_* = 182.22	*F*(000) = 384
Triclinic, *P*1	*D*_x_ = 1.258 Mg m^−^^3^
Hall symbol: -P 1	Mo *K*α radiation, λ = 0.71073 Å
*a* = 9.1383 (3) Å	Cell parameters from 3160 reflections
*b* = 9.5340 (3) Å	θ = 2.4–28.2°
*c* = 12.3138 (4) Å	µ = 0.08 mm^−^^1^
α = 90.794 (2)°	*T* = 173 K
β = 90.528 (2)°	Block, colourless
γ = 116.272 (2)°	0.50 × 0.45 × 0.30 mm
*V* = 961.78 (5) Å^3^	

### Data collection

**Table:**

Bruker APEXII CCD area-detector diffractometer	2809 reflections with *I* > 2σ(*I*)
ω scans	*R*_int_ = 0.023
Absorption correction: multi-scan (*SADABS*; Sheldrick, 1996)	θ_max_ = 25.5°, θ_min_ = 1.7°
*T*_min_ = 0.963, *T*_max_ = 0.978	*h* = −10→11
7592 measured reflections	*k* = −11→11
3498 independent reflections	*l* = −14→14

### Refinement

**Table:**

Refinement on *F*^2^	0 constraints
Least-squares matrix: full	H-atom parameters constrained
*R*[*F*^2^ > 2σ(*F*^2^)] = 0.038	*w* = 1/[σ^2^(*F*_o_^2^) + (0.0414*P*)^2^ + 0.2024*P*] where *P* = (*F*_o_^2^ + 2*F*_c_^2^)/3
*wR*(*F*^2^) = 0.097	(Δ/σ)_max_ < 0.001
*S* = 1.04	Δρ_max_ = 0.19 e Å^−^^3^
3498 reflections	Δρ_min_ = −0.15 e Å^−^^3^
254 parameters	Extinction correction: *SHELXL97* (Sheldrick, 2008), Fc^*^=kFc[1+0.001xFc^2^λ^3^/sin(2θ)]^-1/4^
0 restraints	Extinction coefficient: 0.027 (2)

### Fractional atomic coordinates and isotropic or equivalent isotropic displacement parameters (Å^2^)

**Table:**

	*x*	*y*	*z*	*U*_iso_*/*U*_eq_	
C1A	−0.10944 (18)	0.17265 (18)	0.14642 (13)	0.0397 (4)	
H1A1	−0.1299	0.1843	0.2241	0.048*	
H1A2	−0.211	0.0937	0.1114	0.048*	
C2A	−0.0463 (2)	0.32983 (19)	0.08862 (13)	0.0447 (4)	
H2A1	−0.018	0.4164	0.1424	0.054*	
H2A2	−0.1311	0.3303	0.0382	0.054*	
C3A	0.10660 (19)	0.35042 (18)	0.02512 (13)	0.0419 (4)	
H3A1	0.0836	0.3383	−0.0541	0.05*	
H3A2	0.1987	0.4544	0.0405	0.05*	
C4A	0.14316 (17)	0.22297 (16)	0.06621 (11)	0.0323 (3)	
C5A	0.25796 (16)	0.16684 (15)	0.05611 (11)	0.0307 (3)	
C6A	0.20402 (16)	0.02878 (15)	0.12165 (11)	0.0296 (3)	
C7A	0.26457 (17)	−0.07929 (17)	0.14459 (12)	0.0358 (3)	
H7A	0.3632	−0.0697	0.1133	0.043*	
C8A	0.17821 (18)	−0.20006 (17)	0.21350 (13)	0.0407 (4)	
H8A	0.2178	−0.2746	0.2291	0.049*	
C9A	0.03390 (18)	−0.21502 (18)	0.26077 (12)	0.0422 (4)	
H9A	−0.0218	−0.2986	0.3087	0.051*	
C10A	−0.02975 (18)	−0.11093 (17)	0.23936 (12)	0.0375 (4)	
H10A	−0.1283	−0.1214	0.2712	0.045*	
C11A	0.05677 (16)	0.00985 (16)	0.16923 (11)	0.0304 (3)	
C12A	0.40232 (18)	0.23407 (17)	−0.00564 (12)	0.0361 (3)	
N1A	0.02493 (14)	0.13034 (13)	0.13356 (9)	0.0325 (3)	
N2A	0.52063 (17)	0.28700 (16)	−0.05460 (11)	0.0503 (4)	
C1B	0.49796 (18)	0.56982 (17)	0.73859 (12)	0.0365 (3)	
H1B1	0.6163	0.6215	0.7237	0.044*	
H1B2	0.4801	0.5177	0.8096	0.044*	
C2B	0.4245 (2)	0.68616 (19)	0.73518 (13)	0.0463 (4)	
H2B1	0.5104	0.7941	0.7484	0.056*	
H2B2	0.3405	0.6617	0.7915	0.056*	
C3B	0.34765 (17)	0.67027 (16)	0.62107 (12)	0.0346 (3)	
H3B1	0.2426	0.6768	0.6243	0.041*	
H3B2	0.4221	0.7519	0.5724	0.041*	
C4B	0.32309 (15)	0.51204 (15)	0.58477 (11)	0.0281 (3)	
C5B	0.24306 (16)	0.40067 (15)	0.50510 (11)	0.0297 (3)	
C6B	0.27990 (16)	0.27070 (15)	0.52490 (11)	0.0292 (3)	
C7B	0.23096 (18)	0.12242 (16)	0.47682 (12)	0.0375 (4)	
H7B	0.1594	0.0902	0.4153	0.045*	
C8B	0.28912 (19)	0.02423 (17)	0.52084 (13)	0.0422 (4)	
H8B	0.2565	−0.0768	0.4891	0.051*	
C9B	0.39485 (19)	0.06978 (17)	0.61108 (13)	0.0406 (4)	
H9B	0.4336	−0.0004	0.6387	0.049*	
C10B	0.44413 (17)	0.21428 (16)	0.66078 (12)	0.0344 (3)	
H10B	0.5156	0.2452	0.7224	0.041*	
C11B	0.38481 (16)	0.31282 (15)	0.61696 (11)	0.0285 (3)	
C12B	0.14474 (18)	0.41506 (17)	0.42058 (12)	0.0359 (3)	
N1B	0.40605 (13)	0.45930 (12)	0.65134 (9)	0.0282 (3)	
N2B	0.06647 (18)	0.42763 (17)	0.35107 (12)	0.0546 (4)	

### Atomic displacement parameters (Å^2^)

**Table:**

	*U*^11^	*U*^22^	*U*^33^	*U*^12^	*U*^13^	*U*^23^
C1A	0.0365 (8)	0.0434 (9)	0.0459 (9)	0.0241 (7)	−0.0033 (7)	−0.0072 (7)
C2A	0.0552 (10)	0.0451 (9)	0.0449 (9)	0.0325 (8)	−0.0066 (8)	−0.0048 (7)
C3A	0.0459 (9)	0.0358 (8)	0.0463 (9)	0.0202 (7)	−0.0062 (7)	0.0025 (7)
C4A	0.0342 (8)	0.0300 (7)	0.0291 (7)	0.0111 (6)	−0.0051 (6)	−0.0002 (6)
C5A	0.0299 (7)	0.0304 (7)	0.0295 (7)	0.0113 (6)	−0.0013 (6)	0.0010 (6)
C6A	0.0268 (7)	0.0304 (7)	0.0285 (7)	0.0100 (6)	−0.0044 (6)	−0.0013 (6)
C7A	0.0294 (7)	0.0363 (8)	0.0416 (8)	0.0147 (6)	−0.0052 (6)	0.0001 (6)
C8A	0.0386 (8)	0.0353 (8)	0.0490 (9)	0.0172 (7)	−0.0094 (7)	0.0060 (7)
C9A	0.0397 (9)	0.0385 (8)	0.0403 (9)	0.0098 (7)	−0.0012 (7)	0.0101 (7)
C10A	0.0316 (8)	0.0392 (8)	0.0369 (8)	0.0111 (7)	0.0024 (6)	0.0042 (6)
C11A	0.0302 (7)	0.0316 (7)	0.0285 (7)	0.0131 (6)	−0.0028 (6)	−0.0015 (6)
C12A	0.0379 (8)	0.0341 (8)	0.0345 (8)	0.0142 (7)	0.0007 (7)	0.0028 (6)
N1A	0.0317 (6)	0.0331 (6)	0.0344 (6)	0.0160 (5)	0.0001 (5)	−0.0001 (5)
N2A	0.0452 (8)	0.0503 (8)	0.0512 (8)	0.0169 (7)	0.0130 (7)	0.0078 (7)
C1B	0.0389 (8)	0.0356 (8)	0.0350 (8)	0.0169 (7)	−0.0070 (6)	−0.0070 (6)
C2B	0.0563 (10)	0.0428 (9)	0.0467 (9)	0.0287 (8)	−0.0098 (8)	−0.0113 (7)
C3B	0.0345 (8)	0.0295 (7)	0.0429 (8)	0.0171 (6)	0.0002 (6)	−0.0007 (6)
C4B	0.0263 (7)	0.0274 (7)	0.0319 (7)	0.0131 (6)	0.0043 (6)	0.0047 (6)
C5B	0.0272 (7)	0.0297 (7)	0.0304 (7)	0.0111 (6)	−0.0003 (6)	0.0034 (6)
C6B	0.0271 (7)	0.0274 (7)	0.0313 (7)	0.0105 (6)	0.0036 (6)	0.0029 (6)
C7B	0.0376 (8)	0.0310 (8)	0.0381 (8)	0.0102 (6)	−0.0006 (7)	−0.0028 (6)
C8B	0.0489 (9)	0.0264 (7)	0.0503 (9)	0.0158 (7)	0.0057 (8)	−0.0028 (7)
C9B	0.0460 (9)	0.0325 (8)	0.0507 (9)	0.0237 (7)	0.0073 (7)	0.0077 (7)
C10B	0.0342 (8)	0.0351 (8)	0.0379 (8)	0.0188 (6)	0.0019 (6)	0.0047 (6)
C11B	0.0272 (7)	0.0263 (7)	0.0318 (7)	0.0115 (6)	0.0043 (6)	0.0035 (5)
C12B	0.0352 (8)	0.0347 (8)	0.0375 (8)	0.0153 (7)	−0.0025 (7)	0.0012 (6)
N1B	0.0285 (6)	0.0279 (6)	0.0297 (6)	0.0139 (5)	−0.0022 (5)	−0.0002 (5)
N2B	0.0565 (9)	0.0606 (9)	0.0494 (9)	0.0287 (8)	−0.0155 (7)	0.0012 (7)

### Geometric parameters (Å, º)

**Table:**

C1A—N1A	1.4616 (17)	C1B—N1B	1.4624 (17)
C1A—C2A	1.535 (2)	C1B—C2B	1.530 (2)
C1A—H1A1	0.99	C1B—H1B1	0.99
C1A—H1A2	0.99	C1B—H1B2	0.99
C2A—C3A	1.544 (2)	C2B—C3B	1.540 (2)
C2A—H2A1	0.99	C2B—H2B1	0.99
C2A—H2A2	0.99	C2B—H2B2	0.99
C3A—C4A	1.4891 (19)	C3B—C4B	1.4852 (18)
C3A—H3A1	0.99	C3B—H3B1	0.99
C3A—H3A2	0.99	C3B—H3B2	0.99
C4A—N1A	1.3517 (18)	C4B—N1B	1.3552 (16)
C4A—C5A	1.3784 (19)	C4B—C5B	1.3767 (19)
C5A—C12A	1.419 (2)	C5B—C12B	1.4161 (19)
C5A—C6A	1.4455 (19)	C5B—C6B	1.4437 (19)
C6A—C7A	1.3990 (19)	C6B—C7B	1.4005 (19)
C6A—C11A	1.4104 (19)	C6B—C11B	1.4114 (19)
C7A—C8A	1.380 (2)	C7B—C8B	1.379 (2)
C7A—H7A	0.95	C7B—H7B	0.95
C8A—C9A	1.396 (2)	C8B—C9B	1.397 (2)
C8A—H8A	0.95	C8B—H8B	0.95
C9A—C10A	1.381 (2)	C9B—C10B	1.378 (2)
C9A—H9A	0.95	C9B—H9B	0.95
C10A—C11A	1.3896 (19)	C10B—C11B	1.3882 (18)
C10A—H10A	0.95	C10B—H10B	0.95
C11A—N1A	1.3800 (17)	C11B—N1B	1.3818 (17)
C12A—N2A	1.1508 (19)	C12B—N2B	1.1515 (18)
			
N1A—C1A—C2A	102.51 (12)	N1B—C1B—C2B	101.64 (11)
N1A—C1A—H1A1	111.3	N1B—C1B—H1B1	111.4
C2A—C1A—H1A1	111.3	C2B—C1B—H1B1	111.4
N1A—C1A—H1A2	111.3	N1B—C1B—H1B2	111.4
C2A—C1A—H1A2	111.3	C2B—C1B—H1B2	111.4
H1A1—C1A—H1A2	109.2	H1B1—C1B—H1B2	109.3
C1A—C2A—C3A	107.49 (12)	C1B—C2B—C3B	106.64 (12)
C1A—C2A—H2A1	110.2	C1B—C2B—H2B1	110.4
C3A—C2A—H2A1	110.2	C3B—C2B—H2B1	110.4
C1A—C2A—H2A2	110.2	C1B—C2B—H2B2	110.4
C3A—C2A—H2A2	110.2	C3B—C2B—H2B2	110.4
H2A1—C2A—H2A2	108.5	H2B1—C2B—H2B2	108.6
C4A—C3A—C2A	103.49 (12)	C4B—C3B—C2B	102.46 (11)
C4A—C3A—H3A1	111.1	C4B—C3B—H3B1	111.3
C2A—C3A—H3A1	111.1	C2B—C3B—H3B1	111.3
C4A—C3A—H3A2	111.1	C4B—C3B—H3B2	111.3
C2A—C3A—H3A2	111.1	C2B—C3B—H3B2	111.3
H3A1—C3A—H3A2	109	H3B1—C3B—H3B2	109.2
N1A—C4A—C5A	109.25 (12)	N1B—C4B—C5B	109.20 (11)
N1A—C4A—C3A	110.47 (12)	N1B—C4B—C3B	110.27 (11)
C5A—C4A—C3A	140.26 (13)	C5B—C4B—C3B	140.53 (12)
C4A—C5A—C12A	126.19 (12)	C4B—C5B—C12B	125.28 (13)
C4A—C5A—C6A	106.83 (12)	C4B—C5B—C6B	106.93 (11)
C12A—C5A—C6A	126.98 (13)	C12B—C5B—C6B	127.78 (13)
C7A—C6A—C11A	118.89 (12)	C7B—C6B—C11B	118.82 (12)
C7A—C6A—C5A	134.72 (13)	C7B—C6B—C5B	134.60 (13)
C11A—C6A—C5A	106.40 (12)	C11B—C6B—C5B	106.53 (11)
C8A—C7A—C6A	118.66 (14)	C8B—C7B—C6B	118.46 (14)
C8A—C7A—H7A	120.7	C8B—C7B—H7B	120.8
C6A—C7A—H7A	120.7	C6B—C7B—H7B	120.8
C7A—C8A—C9A	121.37 (14)	C7B—C8B—C9B	121.55 (13)
C7A—C8A—H8A	119.3	C7B—C8B—H8B	119.2
C9A—C8A—H8A	119.3	C9B—C8B—H8B	119.2
C10A—C9A—C8A	121.45 (14)	C10B—C9B—C8B	121.40 (13)
C10A—C9A—H9A	119.3	C10B—C9B—H9B	119.3
C8A—C9A—H9A	119.3	C8B—C9B—H9B	119.3
C9A—C10A—C11A	117.06 (14)	C9B—C10B—C11B	117.09 (14)
C9A—C10A—H10A	121.5	C9B—C10B—H10B	121.5
C11A—C10A—H10A	121.5	C11B—C10B—H10B	121.5
N1A—C11A—C10A	130.29 (13)	N1B—C11B—C10B	130.31 (13)
N1A—C11A—C6A	107.14 (11)	N1B—C11B—C6B	106.98 (11)
C10A—C11A—C6A	122.57 (13)	C10B—C11B—C6B	122.67 (13)
N2A—C12A—C5A	178.68 (16)	N2B—C12B—C5B	179.15 (17)
C4A—N1A—C11A	110.38 (11)	C4B—N1B—C11B	110.34 (11)
C4A—N1A—C1A	114.62 (11)	C4B—N1B—C1B	113.89 (11)
C11A—N1A—C1A	134.86 (12)	C11B—N1B—C1B	135.75 (11)
			
N1A—C1A—C2A—C3A	11.78 (15)	N1B—C1B—C2B—C3B	−21.63 (16)
C1A—C2A—C3A—C4A	−10.85 (16)	C1B—C2B—C3B—C4B	21.44 (16)
C2A—C3A—C4A—N1A	5.68 (16)	C2B—C3B—C4B—N1B	−13.05 (15)
C2A—C3A—C4A—C5A	−175.95 (17)	C2B—C3B—C4B—C5B	166.94 (17)
N1A—C4A—C5A—C12A	−178.53 (13)	N1B—C4B—C5B—C12B	−179.62 (13)
C3A—C4A—C5A—C12A	3.1 (3)	C3B—C4B—C5B—C12B	0.4 (3)
N1A—C4A—C5A—C6A	0.56 (15)	N1B—C4B—C5B—C6B	0.12 (15)
C3A—C4A—C5A—C6A	−177.82 (17)	C3B—C4B—C5B—C6B	−179.87 (16)
C4A—C5A—C6A—C7A	179.59 (15)	C4B—C5B—C6B—C7B	176.58 (15)
C12A—C5A—C6A—C7A	−1.3 (3)	C12B—C5B—C6B—C7B	−3.7 (3)
C4A—C5A—C6A—C11A	−0.40 (14)	C4B—C5B—C6B—C11B	−0.84 (15)
C12A—C5A—C6A—C11A	178.68 (13)	C12B—C5B—C6B—C11B	178.89 (13)
C11A—C6A—C7A—C8A	−0.4 (2)	C11B—C6B—C7B—C8B	−0.8 (2)
C5A—C6A—C7A—C8A	179.65 (14)	C5B—C6B—C7B—C8B	−177.95 (15)
C6A—C7A—C8A—C9A	−0.5 (2)	C6B—C7B—C8B—C9B	−0.2 (2)
C7A—C8A—C9A—C10A	0.9 (2)	C7B—C8B—C9B—C10B	0.7 (2)
C8A—C9A—C10A—C11A	−0.4 (2)	C8B—C9B—C10B—C11B	−0.3 (2)
C9A—C10A—C11A—N1A	−179.53 (14)	C9B—C10B—C11B—N1B	176.69 (13)
C9A—C10A—C11A—C6A	−0.5 (2)	C9B—C10B—C11B—C6B	−0.7 (2)
C7A—C6A—C11A—N1A	−179.89 (12)	C7B—C6B—C11B—N1B	−176.67 (12)
C5A—C6A—C11A—N1A	0.10 (14)	C5B—C6B—C11B—N1B	1.24 (14)
C7A—C6A—C11A—C10A	0.9 (2)	C7B—C6B—C11B—C10B	1.2 (2)
C5A—C6A—C11A—C10A	−179.10 (13)	C5B—C6B—C11B—C10B	179.14 (12)
C5A—C4A—N1A—C11A	−0.52 (15)	C5B—C4B—N1B—C11B	0.68 (15)
C3A—C4A—N1A—C11A	178.38 (11)	C3B—C4B—N1B—C11B	−179.33 (11)
C5A—C4A—N1A—C1A	−176.82 (11)	C5B—C4B—N1B—C1B	179.18 (11)
C3A—C4A—N1A—C1A	2.08 (16)	C3B—C4B—N1B—C1B	−0.83 (16)
C10A—C11A—N1A—C4A	179.36 (14)	C10B—C11B—N1B—C4B	−178.89 (14)
C6A—C11A—N1A—C4A	0.25 (15)	C6B—C11B—N1B—C4B	−1.21 (15)
C10A—C11A—N1A—C1A	−5.4 (3)	C10B—C11B—N1B—C1B	3.1 (3)
C6A—C11A—N1A—C1A	175.50 (14)	C6B—C11B—N1B—C1B	−179.25 (14)
C2A—C1A—N1A—C4A	−8.84 (16)	C2B—C1B—N1B—C4B	14.35 (16)
C2A—C1A—N1A—C11A	176.06 (14)	C2B—C1B—N1B—C11B	−167.66 (15)

### Hydrogen-bond geometry (Å, º)

Cg1 and Cg2 are the centroids of the C6*A*–C11*A* and C6*B*–C11*B* rings, respectively.

**Table:**

*D*—H···*A*	*D*—H	H···*A*	*D*···*A*	*D*—H···*A*
C1*A*—H1*A*1···N2*B*	0.99	2.68	3.338 (2)	124
C2*A*—H2*A*1···N2*B*	0.99	2.66	3.373 (2)	129
C3*A*—H3*A*2···N2*A*^i^	0.99	2.66	3.634 (2)	168
C3*B*—H3*B*1···N2*B*^ii^	0.99	2.57	3.495 (2)	156
C3*A*—H3*A*1···*Cg*1^iii^	0.99	2.79	3.545 (2)	135
C3*B*—H3*B*2···*Cg*2^iv^	0.99	2.67	3.523 (2)	146

Symmetry codes: (i) −*x*+1, −*y*+1, −*z*; (ii) −*x*, −*y*+1, −*z*+1; (iii) −*x*, −*y*, −*z*; (iv) −*x*+1, −*y*+1, −*z*+1.
